# Whole Exome Sequencing Identifies a Novel and a Recurrent Mutation in *BBS2* Gene in a Family with Bardet-Biedl Syndrome

**DOI:** 10.1155/2015/524754

**Published:** 2015-05-11

**Authors:** Yong Mong Bee, Mayank Chawla, Yi Zhao

**Affiliations:** ^1^Department of Endocrinology, Singapore General Hospital, Singapore 169856; ^2^Department of Internal Medicine, Singapore General Hospital, Singapore 169856; ^3^Department of Clinical Research, Singapore General Hospital, Singapore 169856

## Abstract

Bardet-Biedl syndrome (BBS) is a rare autosomal recessive disorder known to be caused by mutations in at least 19 BBS genes. We report the genetic analysis of a patient with indisputable features of BBS including cardinal features such as postaxial polydactyly, retinitis pigmentosa, obesity, and kidney failure. Taking advantage of next-generation sequencing technology, we applied whole exome sequencing (WES) with Sanger direct sequencing to the proband and her unaffected mother. A pair of heterozygous nonsense mutations in *BBS2* gene was identified in the proband, one being novel and the other recurrent. The novel mutation, p.Y644X, resides in exon 16 and was also found in the heterozygous state in the mother. This mutation is not currently found in the dsSNP and 1000 Genome SNP databases and is predicted to be disease causing by *in silico* analysis. This study highlights the potential for a rapid and precise detection of disease causing gene using WES in genetically heterogeneous disorders such as BBS.

## 1. Introduction

Bardet-Biedl syndrome (BBS) is an autosomal recessive ciliopathy with multisystem involvement. The classical clinical picture consists of major diagnostic criteria such as postaxial polydactyly, retinitis pigmentosa, obesity, kidney alteration, genital anomalies, and behavioral dysfunction [1]. Minor diagnostic criteria include diabetes mellitus, ataxia, anosmia, and developmental abnormalities such as Hirschsprung's disease, cardiac malformations, and dental anomalies. Clinical diagnosis is established if four major features or three major and two minor features are present in a patient [1].

To date, 19 BBS genes have been identified and the majority of them are essential for the function of BBSome, a protein complex involved in transporting membrane proteins into and from cilia [2, 3]. Mutations in the BBS genes account for more than 80% of BBS patients while 20% of them still lack molecular diagnosis [4]. The large number of genes makes diagnosis of BBS by direct deoxyribonucleic acid (DNA) sequencing both time-consuming and costly. Recently, the advent of next-generation sequencing technologies accelerated the identification of novel BBS genes and causative disease mutations in known genes [5].

In this study, we aimed to determine the genetic cause of BBS in a patient from a nonconsanguineous family using whole exome sequencing (WES).

## 2. Subjects and Methods

### 2.1. Subjects

**Figure 1:**
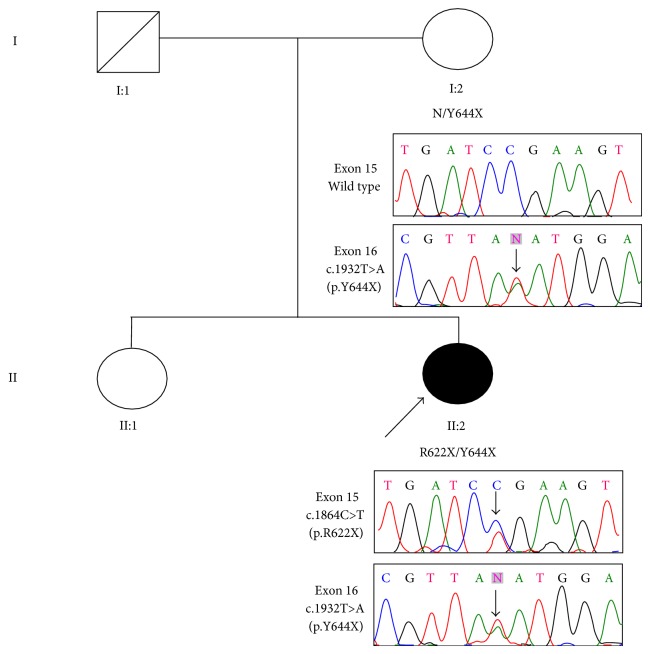
Pedigree chart of our patient's family with BBS. Results of Sanger sequencing for both the proband and her mother are shown. Father and sister's DNA were unavailable. Filled and unfilled symbols indicate affected and unaffected individuals, respectively. Squares and circles represent males and females, respectively. The arrow indicates the proband.

The study was approved by the ethics committee/institutional review board of Singapore Health Services Pte Ltd. Written informed consent was obtained from both the proband (II:2) and her mother (I:2) before the study began (Figure 1). The father (I:1) had demised and the sister (II:1) was residing overseas during the time of the study. Blood samples from the proband and her mother were collected and processed.

### 2.2. Genomic DNA Extraction and Exome Sequencing

Genomic DNA was extracted from the proband and her mother and purified from peripheral blood leucocytes using the QIAamp blood kit according to the manufacturer's protocols (QIAGEN, Hilden, Germany). WES was performed by Macrogen, Korea. Exons of DNA samples were captured using the in-solution SureSelect Target Enrichment System (Agilent, Human All Exon Kits v2) (Agilent Technologies, Inc., Santa Clara, CA, USA) followed by a paired-end high-throughput sequencing on reads of 75 bp using Illumina HiSeq 2000 (Illumine Inc., San Diego, CA, USA). Image analysis was performed with default parameters of Illumina RTA v1.12.4 pipeline. Base calling was performed with CASAVA 1.8.2 (Illumina).

### 2.3. Alignment, Variant Calling, and Mutation Detection

The sequence reads were aligned with the human genome reference sequence (University of California Santa Cruz, human genome assembly 19 (UCSC hg19)) using the Burrows-Wheeler transform algorithm. Variants (single-nucleotide variants (SNVs) and short insertion-deletion variants (Indels)) were called with SAMTools software (http://samtools.sourceforge.net/) with reference to public databases dbSNP and 1000 Genomes. SNVs were filtered for a minimum Phred quality score of 30, allowing 99.9% base call accuracy. Analysis was performed with preference given to variants located in the 19 genes causing BBS. We first removed nonexonic variants and variants indicated as synonymous. This was followed by the removal of common variants (i.e., minor allele frequency > 0.01) reported in public databases. SIFT, PolyPhen-2, and MutationTaster were used for prediction of functional effects of the selected variants. All missense variants predicted to be benign were removed from the list of variants. Within the prioritized variants, those harboring truncating mutations or mutations predicted to be damaging were considered to be the most promising candidates. PubMed and OMIM were reviewed for previous publications regarding candidate genes and functional and expression data.

### 2.4. Sanger Sequencing

Sanger sequencing using standard polymerase chain reaction (PCR) amplification procedures was performed for confirmation of candidate variants seen in WES as well as segregation of candidate variants within the family. Primers and PCR conditions are available on request.

## 3. Results

### 3.1. Clinical Features

The proband (II-2) was a 37-year-old woman born from nonconsanguineous parents of Malay ethnicity. She had postaxial polydactyly over her left hand and both feet at birth for which she underwent corrective surgery at the age of five. She started to experience declining vision from the age of five secondary to retinitis pigmentosa. She became legally blind at the age of 10. She developed obesity in early childhood and her mother recalled the proband as a child with an insatiable appetite. She also had learning difficulties for which she attended a junior school for special needs children followed by a vocational school.

As an adult, she was obese with a body mass index of 31.0 kg/m^2^. She was diagnosed with diabetes mellitus at the age of 19 following her presentation with osmotic symptoms. Both the glutamic acid decarboxylase and islet cell antibodies were negative. She was treated with glipizide and her latest HbA1c was 6.6%. She presented with proteinuria at the age of 30. This was associated with a gradual decline in her renal function and she developed renal failure at the age of 34. She also had dental anomalies in the form of dental crowding. She was diagnosed with BBS based on four major features (polydactyly, retinitis pigmentosa, obesity, and renal failure) and two minor features (diabetes mellitus and dental anomalies).

The proband has an elder sister (II:1) who was unaffected. Her father (I:1) passed away from suspected cancer in his 60s. Her mother was recently diagnosed with type 2 diabetes mellitus. None of her extended family members were diagnosed with BBS.

### 3.2. Mutation Detection

We applied WES to the proband and her mother. Coverage of targeted regions for both samples was more than 98.9% with an average sequencing depth of 64.8. Targeted regions with greater than 10 times of coverage were 95.3% and 97.2%, respectively. WES yielded a total of 70,312 and 73,290 variants in the proband (II:2) and her mother (I:2), respectively (Table 1).

Following the step-by-step filtering protocol described above, two heterozygous nonsense mutations in* BBS2* were finalized in the proband. One* BBS2* mutation was a single base pair substitution leading to a nonsense mutation c.1864C>T (p.R622X) in exon 15, initiating a premature stop codon in the protein. MutationTaster predicts this variant to be disease causing by protein truncation and nonsense-mediated decay. This mutation has been previously reported in a BBS family with compound heterozygous mutations in the* BBS2* gene [6].

**Figure 2:**
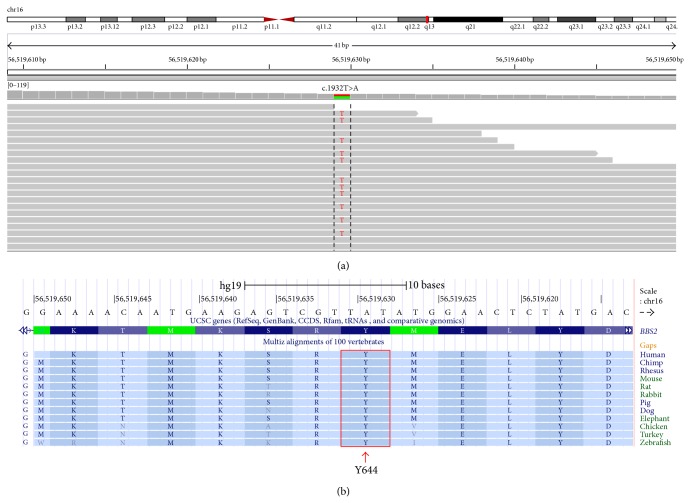
(a) Excerpt of whole exome sequencing data visualized in the Integrative Genomics Viewer. The novel nonsense mutation in exon 16 of* BBS2* is shown in the proband. The figure displays the forward strand. (b) The panel from the UCSC genome browser (http://genome.ucsc.edu/) shows multispecies alignment to highlight the strong conservation of the affected Y644 residue.

The second* BBS2* mutation was a nonsense mutation c.1932T>A (p.Y644X) in exon 16. MutationTaster predicts this variant to be disease causing as well. This mutation was also present in the mother in a heterozygous state. This mutation has not been previously reported and was not observed in the dsSNP and 1000 Genome SNPs. It was not also found in the Exome Variant Server (http://evs.gs.washington.edu/EVS/) database either. The residue affected is highly conserved across multiple species (Figure 2).

To confirm the WES results, we performed Sanger sequencing to validate the mutation in this family. In the proband, the compound heterozygous mutations were confirmed. The unaffected mother was found to be heterozygous for the c.1932T>A (p.Y644X) mutation (Figure 1).

## 4. Discussion

In this study, WES revealed a pair of nonsense mutations in* BBS2*, one of which is novel. We also showed that the combination of WES and Sanger sequencing can be an effective approach for molecular diagnosis of an extremely heterogeneous disorder such as BBS.

To date, 19 BBS causing genes have been identified [2, 3]. A common feature of BBS proteins is that they are involved in ciliary function and intraflagellar transport (IFT). Seven of the BBS proteins (BBS1, BBS2, BBS4, BBS5, BBS7, BBS8, and BBS9) have been shown to form a complex known as the BBSome [7], which appears to play a role in ciliogenesis. In particular, BBS2 plays an important role as an intermediate complex in the assembly of the mature BBSome. It directly interacts with BBS7 and BBS9 to form the BBSome core complex, which is required for complete formation of the BBSome [8]. Hence, BBS2 mutations are detrimental to the ciliogenesis process. The BBS3 is required for ciliary localization of the BBSome and BBS6, BBS10, and BBS12 act as chaperonins and mediate BBSome assembly [9, 10].

The gene* BBS2*, located on chromosome 16q21, consists of 18 exons and encodes a 721-amino-acid protein. The gene contains a 45-amino-acid coiled coin region within exons 9 and 10 and this is flanked by peptide chain regions on either side [11].* BBS2* carries the third largest mutational load (12.0%), after* BBS10* (21.7%) and* BBS1* (16.9%) [6]. In this study, two nonsense mutations in* BBS2* were identified in the proband. The first mutation in exon 15, c.1864C>T (p.R622X), was previously reported in a pedigree of South African Black/European/Asian ancestry [6]. Since the father (I:1) was phenotypically normal, it is thus conceivable that he carried this mutation in a heterozygous state and transmitted it to the proband. The second mutation in exon 16, c.1932T>A (p.Y644X), is present in both the proband and her mother (I:2). This variant has not been reported previously [6]. This nonsense mutation is located within a highly conserved region compared to different species (Figure 2). To date, more than 10% of reported mutations in* BBS2* are located in exons 2, 4, and 6 while only 6% were located in exons 15 and 16 [12].

In this study, the proband had obesity and diabetes mellitus. Obesity is a cardinal feature of BBS and diabetes has historically been considered as a minor feature of BBS. Given the close association between obesity and insulin resistance, a large proportion of obese BBS patients would be expected to have glucose intolerance. However, type 2 diabetes mellitus is not a common feature of BBS. Overt diabetes was reported in only 6% of a cohort of 33 BBS patients, whereas obesity was present in 70% of the same cohort [13]. It is even more surprising that BBS gene inactivation has been shown to be protective against diabetes [14]. In vitro studies on human primary mesenchymal stem cells (MSCs) showed that* BBS12* inactivation facilitated adipogenesis, increased insulin sensitivity, increased glucose utilisation, and decreased inflammation in the fat. It has been proposed that the apparent discrepancy observed in this condition could be due to phenotypic variation as it has been recognized that not all individuals with an identical mutation are equally affected. Interacting genetic variants may also have given rise to different degree of dysfunction [14].

The advent of high-throughput sequencing in recent years has changed the molecular diagnostic protocols for disorders with considerable clinical and genetic heterogeneity such as BBS. Our work demonstrates the application of WES to a precise genetic testing in a BBS pedigree within a short time frame. One could argue that, by adopting Sanger method of candidate gene sequencing,* BBS2* mutations would have been identified successfully in this case. However, PCR amplification of each candidate gene, because of the great breath of coding regions, is cumbersome, time-consuming, and potentially not cost-efficient. Exome sequencing has the advantage of being less biased and saves time compared to candidate gene sequencing. With falling costs, this approach will likely be the more cost effective way of genetic diagnosis of rare Mendelian diseases. Besides the great value of WES in finding a mutation in known genes, it is also capable of identifying mutations in novel genes. The recent discovery of novel disease causing genes for BBS (i.e.,* LZTFL1*,* BBIP1*, and* IFT27*) was secondary to the adoption of WES in the diagnostic pathways [2, 3, 15]. While WES is clearly more exhaustive in gene coverage, researchers have to be mindful that 20–30% of targeted exons are not sufficiently covered for diagnostic accuracy. This may pose a potential risk of false negative findings when adopting this technology for routine diagnosis in clinical practice.

## 5. Conclusions

In our study, we have elucidated the genetic cause of BBS in our patient. A novel (p.Y644X) and a recurrent (p.R622X) nonsense mutation in* BBS2* were identified in the proband. We have also demonstrated the usefulness of WES of a small number of family members in identifying the responsible gene. This molecular diagnostic approach will likely be incorporated into routine clinical evaluation of patients with suspected genetic disorders like BBS.

## Figures and Tables

**Table 1:** Summary of variants detected through WES.

	Proband (II:2)	Mother (I:2)
Total number of variants obtained	70,312	73,290
Exonic nonsynonymous variants	9,901	9,964
Exonic nonsynonymous variants (MAF <0.01)	1,154	1,182
Nonsynonymous SNP	664	648
In-frame coding indel	224	269
Frame shift	73	45
Nonsense	24	11
Read through	1	1
^#^BBS causing variants	2	1

^#^Analysis was performed with preference given to those variants that were located in the 19 genes causing BBS.

WES: whole exome sequencing; MAF: minor allele frequency; SNP: single-nucleotide polymorphism; BBS: Bardet-Biedl syndrome.
